# Pilocarpine-Induced Status Epilepticus Is Associated with Changes in the Actin-Modulating Protein Synaptopodin and Alterations in Long-Term Potentiation in the Mouse Hippocampus

**DOI:** 10.1155/2017/2652560

**Published:** 2017-01-05

**Authors:** Maximilian Lenz, Marina Ben Shimon, Thomas Deller, Andreas Vlachos, Nicola Maggio

**Affiliations:** ^1^Department of Neurology, The Chaim Sheba Medical Center, Tel HaShomer, Israel; ^2^Institute of Anatomy II, Faculty of Medicine, Heinrich-Heine University, Düsseldorf, Germany; ^3^Institute of Clinical Neuroanatomy, Neuroscience Center Frankfurt, Goethe University, Frankfurt, Germany; ^4^Sackler Faculty of Medicine, Tel Aviv University, Tel Aviv, Israel; ^5^Talpiot Medical Leadership Program, The Chaim Sheba Medical Center, Tel HaShomer, Israel; ^6^Department of Neurology, Sackler Faculty of Medicine, Tel Aviv University, Tel Aviv, Israel; ^7^Sagol School of Neuroscience, Tel Aviv University, Tel Aviv, Israel

## Abstract

Epilepsy is a complex neurological disorder which can severely affect neuronal function. Some patients may experience status epilepticus, a life-threatening state of ongoing seizure activity associated with postictal cognitive dysfunction. However, the molecular mechanisms by which status epilepticus influences brain function beyond seizure activity remain not well understood. Here, we addressed the question of whether pilocarpine-induced status epilepticus affects synaptopodin (SP), an actin-binding protein, which regulates the ability of neurons to express synaptic plasticity. This makes SP an interesting marker for epilepsy-associated alterations in synaptic function. Indeed, single dose intraperitoneal pilocarpine injection (250 mg/kg) in three-month-old male C57BL/6J mice leads to a rapid reduction in hippocampal SP-cluster sizes and numbers (in CA1 stratum radiatum of the dorsal hippocampus; 90 min after injection). In line with this observation (and previous work using SP-deficient mice), a defect in the ability to induce long-term potentiation (LTP) of Schaffer collateral-CA1 synapses is observed. Based on these findings we propose that status epilepticus could exert its aftereffects on cognition at least in part by perturbing SP-dependent mechanisms of synaptic plasticity.

## 1. Introduction

Epilepsy subsumes a group of serious disorders of the central nervous system characterized by a predisposition to recurrent unprovoked seizures, that is, abnormal excessive synchronous neural activity [1]. Several forms of epilepsy are currently distinguished, with temporal lobe epilepsy being among the most resistant ones to pharmacological treatment [2, 3]. If not treated (or not responding to medication), some patients with epilepsy will experience an episode of prolonged seizure activity, status epilepticus (SE), which is a life-threatening condition [4, 5]. While SE represents an exacerbation or even initial manifestation of epileptic disorders, it can also result from other brain pathologies, such as traumatic brain injury or stroke [6, 7]. Although aggressive treatment may halt SE, surviving patients experience severe postictal cognitive dysfunctions, such as memory deficits and spatial/temporal disorientation [2, 8–11].

Animal models of SE have been employed to demonstrate that ongoing epileptic activity in the brain can affect both excitatory and inhibitory synapses and thus neuronal plasticity [12–15]. However, the molecular mechanisms and cellular targets through which SE affects synaptic plasticity remain not well understood. Here, we employed the well-established model of pilocarpine-induced status epilepticus, which can be linked to alterations in memory performance [11], to study the effects of SE on synaptopodin (SP [16]) and synaptic plasticity.

SP is an actin-modulating protein expressed in cortical principal neurons [16]. It is a marker and essential component for the spine apparatus organelle and has been firmly linked to the ability of neurons to express synaptic plasticity (e.g., [17–19]). Hence, in an attempt to establish SP as a marker for epilepsy-associated alterations in synaptic plasticity, we tested whether SE-induced alterations in synaptic plasticity (e.g., [20]) are accompanied by* in vivo* changes of SP.

## 2. Materials and Methods

### 2.1. Animals and Seizure Staging

**Figure 1:**
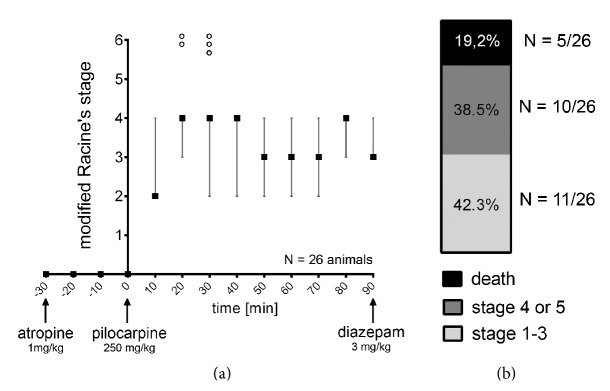
Intraperitoneal pilocarpine injection leads to stable behavioral seizure activity. (a) After a single dose of intraperitoneal pilocarpine injection (250 mg/kg), behavioral seizure activity was documented every 10 minutes using the modified Racine's stages. Stage 6 animals (indicated by black circles) were not included in the analysis.(stage 0 = no seizures, 1 = freezing, 2 = single twitches, 3 = orofacial seizures, 4 = clonic seizures, 5 = tonic seizures, and 6 = death). (b) 90 minutes after pilocarpine injection 42.3% of the animals were in stages 1–3 and 38.5% were in stage 4 or 5 of the modified Racine's stages. 5 out of 26 animals died during experimental procedure and were not included in further analysis (*N* = 26 animals; stages 1–3: 11/26 animals, stage 4 or 5: 10/26 animals, death: 5/26 animals).

Animal handling was approved by the Institutional Animal Care and Use Committee at the Chaim Sheba Medical Center, which adheres to the national law and NIH rules. Briefly, SE was induced in 3-month-old male C57BL/6J mice by a single intraperitoneal (ip) injection of 250 mg/kg pilocarpine hydrochloride. In order to avoid side effects induced by peripheral cholinergic activation, mice were treated with atropine sulphate monohydrate (1 mg/kg, ip) 30 minutes before pilocarpine injection, while diazepam (3 mg/kg; ip) was used to halt convulsions prior to experimental assessment (90 min after pilocarpine injection; Figure 1(a)). After pilocarpine injection, behavioral seizure activity was documented every 10 minutes by an investigator blind to experimental conditions using the modified Racine's stages (c.f. [20]; 0 = no seizures, 1 = freezing, 2 = single twitches, 3 = orofacial seizures, 4 = clonic seizures, 5 = tonic seizures, and 6 = death). Control groups received atropine, diazepam, and vehicle-only (instead of pilocarpine). Animals were subjected to further experimental assessment 3–5 min after diazepam injection.

### 2.2. Immunostaining and Microscopy

Deeply anaesthetized mice were transcardially perfused with 4% (w/v) paraformaldehyde in phosphate buffered saline (PBS, 0.1 M, pH 7.4) for 20 min. Brains were removed and postfixed in the same fixative for at least 24 h. Serial coronal sections (50 *μ*m) were cut with a vibratome (VT 1000S, Leica, Bensheim, Germany) and stained with an antibody against SP as previously described [19, 21]. Briefly, sections containing the dorsal hippocampus were incubated for 1 h with 10% (v/v) normal goat serum (NGS) in 0.5% (v/v) Triton X-100 containing PBS to reduce unspecific staining and subsequently incubated for 48 h at 4°C in rabbit anti-SP antibody (1 : 1000 in 10% (v/v) NGS, 0.1% (v/v) Triton X-100; SE-19, Sigma Aldrich). Sections were washed and incubated for 3 h with Alexa 488-labeled donkey anti-rabbit antibody (1 : 1000, 10% (v/v) NGS, 0.1% (v/v) Triton X-100; Invitrogen). TO-PRO® (Invitrogen) nuclear stain was used to visualize cytoarchitecture (1 : 5000 in PBS; 10 min). Sections were washed again, transferred onto glass microscope slides, and mounted with antifading mounting medium (DAKO Fluoromount). Confocal images were acquired using a Nikon Eclipse C1si laser-scanning microscope equipped with a 60x oil-immersion objective lens (NA 1.4; Nikon). All high resolution images (60x objective lens; 7x scan zoom) were acquired at tissue levels ~5 *μ*m below the surface using the exact same settings at the microscope (detector gain and amplifier were initially set to obtain pixel densities within a linear range).

### 2.3. RNA Extraction and Quantitative PCR (qPCR)

Mice were deeply anaesthetized using ketamine/xylazine (100 mg/kg and 10 mg/kg, resp.) and rapidly decapitated. Whole hippocampi were isolated and immediately frozen in liquid nitrogen. Tissue was transferred into ice-cold TRIzol® and homogenized. RNA was extracted via phenol-chloroform phase separation and eluted with Bio-Rad Aurum 732-6820 kit (Bio-Rad, CA, USA). 1 *μ*g of total RNA was used for reverse transcription using High Capacity cDNA Reverse Transcription Kit (Applied Biosystems, Rhenium, Israel). qPCR was performed using the StepOnePlus™ Real-Time PCR system (Applied Biosystems). Targets were amplified using SYBR Green with Hypoxanthine Guanine Phosphoribosyltransferase as reference gene (HPRT: forward primer: 5′-TGAAAGACTTGCTCGAGATGTCA-3′; reverse primer: 5′-CACACAGAGGGCCACAATGT-3′; SP: forward primer: 5′-GTCTCCTCGAGCCAAGCA-3′; reverse primer: 5′-CACACCTGGGCCTCGAT-3′). A standard qPCR protocol was used: 1 cycle of 50°C for 2 min, 1 cycle of 95°C for 10 min, and 40 cycles of 95°C for 15 s and 60°C for 1 min. The average CT values (mean ± standard deviation) of synaptopodin gene expression in control and SE were 21.65 ± 0.35 and 21.76 ± 0.15, respectively.

### 2.4. Electrophysiology

Mice were deeply anaesthetized with ketamine/xylazine (100 mg/kg and 10 mg/kg, resp.) before rapid decapitation. After removing the brain, 400 *μ*m coronal slices containing the dorsal hippocampus, which expresses robust long-term potentiation under physiological conditions (c.f. [22, 23]), were prepared using a vibratome (World Precision Instruments). Slices were consecutively incubated for 1.5 h in a humidified, carbogenated (5% CO_2_ and 95% O_2_) gas atmosphere at 33 ± 1°C and were superfused with artificial CSF (containing (in mM) 124 NaCl, 2 KCl, 26 NaHCO_3_, 1.24 KH_2_PO_4_, 2.5 CaCl_2_, 2 MgSO_4_, and 10 glucose, pH 7.4) in a standard interface chamber. Recordings were made with a glass pipette containing 0.75 M NaCl (4 MΩ) placed in the stratum radiatum CA1. Stimulation was evoked using a Master 8 pulse stimulator (A.M.P.I., Jerusalem, Israel) and was delivered through a set of bipolar nichrome electrodes placed on a side of the recording electrode. LTP was induced by high-frequency stimulation consisting of 100 pulses at twice the test intensity, delivered at a frequency of 100 Hz (HFS; 100 Hz, 1 s; [24]). Before applying the tetanic stimulation, baseline values were recorded at a frequency of 0.033 Hz. Responses were digitized at 5 kHz and stored on a computer.

### 2.5. Quantification and Statistics

qPCR data were analyzed using HPRT as reference gene. Analysis was performed according to Pfaffl [25]. The qPCR assay efficiency was calculated with the StepOnePlus software (Applied Biosystems, USA) based on dilution series of five samples for each assay. Values were normalized to vehicle-treated controls. Immunolabeled SP-clusters were analyzed in stratum radiatum of area CA1 using the Image J software package (available from http://rsb.info.nih.gov/ij) [26, 27]. SP-clusters were assessed in single plane confocal images by setting the same threshold value and minimal pixel size for all images using the “analyze particles” function of ImageJ software. Three visual fields in CA1 stratum radiatum were analyzed in each slice and SP-cluster sizes and numbers were averaged for each hippocampus. Values were normalized to the results obtained in age- and time-matched vehicle-treated control animals.

Electrophysiological data were analyzed offline using Spike 2 software. Field excitatory postsynaptic potential fEPSP slope changes after tetanic stimulation were calculated with respect to baseline. Statistical comparisons between the two groups were performed using a *t*-test (unpaired, two-tailed). Immunostainings and qPCR analysis were statistically compared using the nonparametric Mann-Whitney test, since normal distribution of these data cannot be assured. *P* values smaller 0.05 were considered a significant difference. In the text and figure values represent mean ± standard error of the mean (SEM), unless indicated otherwise. Values from seizure staging represent median ± interquartile range. ^*∗*^ *P* < 0.05, ^*∗∗*^ *P* < 0.01, and ^*∗∗∗*^ *P* < 0.001; nonsignificant differences are indicated by “NS.”

## 3. Results

Adult 3-month-old C57BL/6J animals were injected with atropine (1 mg/kg; i.p.) 30 min before a single dose of pilocarpine (250 mg/kg; ip) to induce stable SE. Behavioral seizure staging was documented using the modified Racine's stages (Figure 1). Intraperitoneal injection of 250 mg/kg pilocarpine induced rapid-onset and stable behavioral seizure activity (Figure 1(a)). After 90 minutes, 42.3% (11 animals out of 26) of all animals were in stages 1–3 and 38.5% (10 animals out of 26) were in stage 4 or 5 of the modified Racine's stages. 5 out of 26 animals died during experimental procedure (Figure 1(b); these animals were not included in further analysis). After 90 minutes, diazepam was used (3 mg/kg; ip) to halt convulsions prior to experimental assessment. We did not observe any apparent correlation between seizure stage and both effects on LTP-alterations and changes in synaptopodin cluster properties. Age- and time-matched control animals were treated in the same way except for pilocarpine injection, which was replaced by injecting the exact same volume of vehicle-only. Changes in SP were assessed in CA1 stratum radiatum of the dorsal hippocampus and LTP was probed at Schaffer collateral-CA1 synapses to correlate SE-induced changes in SP with alterations in associative synaptic plasticity.

### 3.1. Pilocarpine-Induced Status Epilepticus Decreases Synaptopodin Cluster Sizes and Numbers

**Figure 2:**
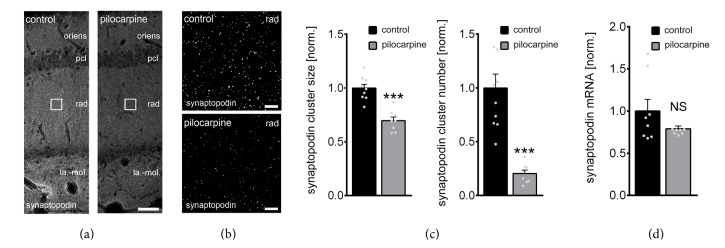
Pilocarpine-induced status epilepticus (SE) affects synaptopodin clusters in CA1 stratum radiatum. (a–c) Synaptopodin cluster sizes and numbers were assessed in CA1 stratum radiatum of anatomically matched coronal sections containing the dorsal hippocampus from pilocarpine- or vehicle-only-treated animals. *N*_control_ = 10 hippocampal slices of 5 animals, *N*_pilocarpine_ = 8 hippocampal slices of 4 animals; both hippocampi in each animal analyzed; averaged values from 3 images obtained from each slice (stratum oriens, oriens; stratum pyramidale, pcl; stratum radiatum, rad; stratum lacunosum-moleculare, la.-mol.). Values normalized to control. Gray dots indicate individual data points (one data point outside the axis limits for control synaptopodin cluster number). Mann-Whitney test; ^*∗∗∗*^ *P* < 0.001. Scale bars, in (a) 50 *μ*m, in (b) 4 *μ*m. (d) Synaptopodin mRNA levels are not significantly changed after single episode of SE. The results are normalized to HPRT gene expression within the same cDNA sample and are represented as the relative levels of the mean ± SEM versus control. *N* = 8 hippocampi from 8 animals of each group. Mann-Whitney test; *P* = 0.63, NS, not significant difference.

**Figure 3:**
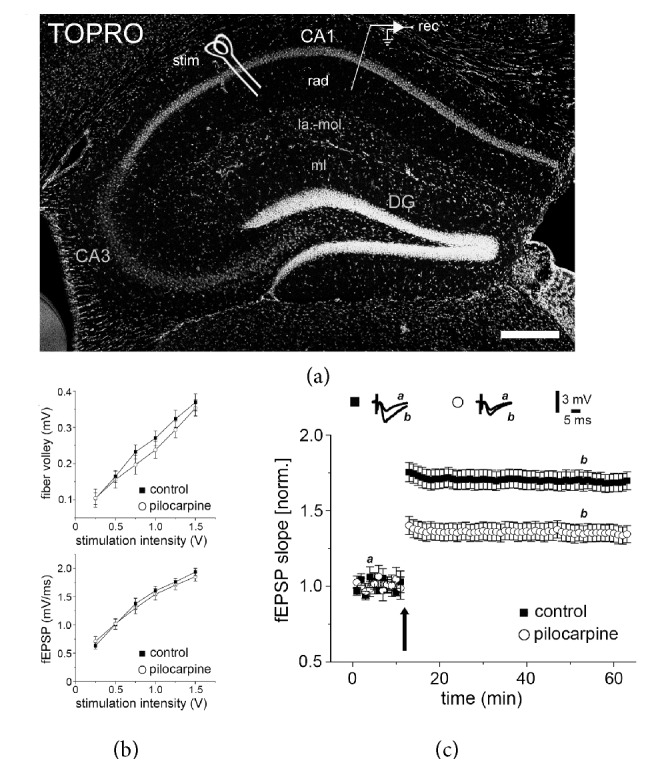
Pilocarpine-induced status epilepticus affects long-term potentiation (LTP) of Schaffer collateral-CA1 synapses. (a–c) LTP of Schaffer collateral-CA1 synapses was probed in acute dorsal hippocampal slices. A single 100 Hz tetanus (1 s) is applied (stim), to induce LTP (recordings of field excitatory postsynaptic potentials; fEPSP) in slices prepared from either pilocarpine- or vehicle-injected animals (stratum radiatum, rad; stratum lacunosum-moleculare, la.-mol.; stratum moleculare, mL; dentate gyrus, DG; nuclear stain, TO-PRO; scale bar: 200 *μ*m). While no significant difference in fiber volleys and input/output properties is detected (b), a deficit in the ability to induce LTP at Schaffer collateral-CA1 synapses is observed in the pilocarpine group (c). Values normalized to baseline fEPSP slope. control_50 min_ = 1.69 ± 0.06, pilocarpine_50 min_ = 1.35 ± 0.05; *P* = 0.001; *N* = 9 slices (1 slice per animal) of each group. *t*-test (unpaired, two-tailed).

Anatomically matched coronal brain slices containing the dorsal hippocampus were immunostained for SP (Figure 2(a)) to assess SE-induced changes in SP-cluster properties. Sizes and numbers of SP-clusters were determined in confocal images of CA1 stratum radiatum in pilocarpine-treated animals and controls (Figure 2(b); cf., LTP of Schaffer collateral-CA1 synapses, Figure 3). Indeed, a significant reduction in both cluster sizes and numbers following pilocarpine-induced status epilepticus was observed in these experiments (Figure 2(c)). Particularly, the decrease in SP-cluster numbers was prominent (−80%). These results disclose that SP-clusters are severely affected after SE-induction.

### 3.2. Synaptopodin mRNA Levels Are Not Changed after Pilocarpine-Induced SE

We next speculated that SE-induced alterations in SP-cluster properties could be accompanied by changes in SP-mRNA levels. Hence, we repeated SE-experiments (and vehicle-injections) in a different set of mice and isolated the hippocampi to assess differences in mRNA levels using qPCR (Figure 2(d)). HPRT served as a reference gene in these experiments. Although a trend toward reduced SP-mRNA levels was observed, the change did not reach the level of significance (Figure 2(d); Mann-Whitney test; *P* = 0.63). We conclude that the SE-induced changes in SP-cluster sizes and numbers are not the result of a strong reduction in SP-mRNA levels.

### 3.3. SE-Induced Changes in Synaptopodin Clusters Reflect SE-Induced Alterations in LTP

Based on our previous work, which disclosed an interdependency between SP and the ability of neurons to express synaptic plasticity [17–19, 21], we hypothesized that SE-induced changes in SP-cluster sizes/numbers will be associated with alterations in the expression of synaptic plasticity. Hence, LTP at Schaffer collateral-CA1 synapses was probed in anatomically matched dorsal hippocampal slices of pilocarpine- and vehicle-injected animals (Figure 3(a)). Notably, baseline synaptic transmission was not affected after SE-induction: both fiber volleys and input/output properties were not significantly different between the two groups (Figure 3(b)). After obtaining stable baseline recordings, tetanic stimulation (1 s; 100 Hz) was used to induce LTP. Indeed, we observed an LTP-defect in SE-treated animals as predicted by the reduction in SP-clusters (Figure 3(c)) and our previous investigations [17, 18, 21]. We conclude that SE-induced changes in SP are accompanied by a deficit in the ability of neurons to express associative synaptic plasticity.

## 4. Discussion

SP has been firmly linked to the ability of neurons to express synaptic plasticity [28–31]: SP-deficient mice exhibit a deficit in the ability to induce LTP [17] as well as homeostatic synaptic plasticity [19]. Moreover, evidence has been provided that SP affects dendritic spine plasticity [18, 30]. Despite these intriguing phenotypes, SP-deficiency seems not to affect spine numbers and baseline synaptic transmission, since both input/output properties of field potential recordings and single cell excitatory synaptic current recordings are not altered in SP-deficient preparations [17, 19, 32]. Hence, SP reflects the ability of neurons to express synaptic plasticity and not basic synaptic properties. This suggestion is supported by the results of the present study on unaltered baseline synaptic transmission after SE-induction (Figure 3(b)), despite severe changes in SP-cluster sizes/numbers in response to a single episode of SE (Figure 2(c)).

The signals which regulate SP-expression and SP-clustering under physiological and pathological conditions remain not well understood. While evidence has been provided that the induction of LTP leads to an NMDA-R dependent increase in SP-mRNA and SP-protein levels [28, 29], an inverse interrelation between synaptic activity and SP has also been demonstrated [19]. In this earlier study prolonged inhibition of NMDA-Rs (or voltage sensitive Ca^2+^-channels) caused an increase in SP-cluster sizes without affecting baseline synaptic transmission [19]. Hence, SP appears to be regulated by Ca^2+^-dependent positive- and negative-feedback mechanisms. Although it remains to be shown how distinct temporal (and spatial) Ca^2+^-signals affect SP, the results of the present study are consistent with a homeostatic, that is, negative-feedback mechanism: prolonged seizure activity leads to a rapid reduction in SP-cluster sizes/numbers. Notably, previous work has demonstrated increased NMDA-R currents following pilocarpine-induced SE, which may support our hypothesis on Ca^2+^-dependent negative-feedback mechanisms [33–35]. In addition, we have recently reported a similar effect on SP-clusters in a model of systemic inflammation using ip injection of lipopolysaccharides (LPS) [21]. Considering that SE-induction has been linked to neuroinflammation [36], it will be important to compare SE- and LPS-induced changes in SP and to determine the role of cytokines in SP-cluster regulation.

The consequences of SE-induced alterations in SP-cluster properties and LTP-induction need to be addressed in future studies. On one hand, alterations in LTP may underlie cognitive dysfunction seen in the context of SE [11, 12]. On the other hand, it is important to also consider that a reduction in the ability of neurons to express LTP may protect the network from maladaptive changes and circuit reverberation. Hence, it will be helpful to better understand whether restoring the ability of neurons to express LTP is detrimental or beneficial for the course of the disease. Thus, it is interesting to speculate that SE-induced alterations in LTP may protect neuronal networks from the development of epilepsy at the expense of cognitive function. However, at this point we have to concede that we do not know if SE-induced alterations in SP-expression are observed also in other brain regions, for example, in the ventral hippocampus, and whether changes in SP strictly reflect only alterations in associative synaptic plasticity [19, 27].

Regardless of these considerations the results of the present study are in line with an earlier report on the effects of kainic acid-induced SE on SP [37]. In this previous study, a semiquantitative assessment of postictal SP-changes in CA1 disclosed no changes in SP-mRNA levels but reduced SP-immunoreactivity upon ip kainic acid-injection. Apparently, SE-induced changes in SP are not only seen in the pilocarpine-induced model of temporal lobe epilepsy.

## 5. Conclusion

The results of the present study demonstrate that SE induces the remodeling of SP-clusters and leads to alterations in associative synaptic plasticity. Based on these results and previous work on the role of SP in synaptic plasticity and behavioral learning [17, 38], we propose that SP could be one of the neuronal targets through which prolonged seizures affect the ability of neurons to express synaptic plasticity. Although more work is required to unravel the precise mechanisms which regulate SP under SE conditions, and to better understand the role of SP (and synaptic plasticity) in epilepsy, we are confident to propose that SP may serve as a marker molecule for seizure-induced alterations in the ability of neurons to express synaptic plasticity, that is, SE-related synaptopathies.
